# Exhaled breath analysis in interstitial lung disease

**DOI:** 10.1097/MCP.0000000000000978

**Published:** 2023-07-05

**Authors:** Iris G. van der Sar, Marlies S. Wijsenbeek, Catharina C. Moor

**Affiliations:** Department of Respiratory Medicine, Erasmus University Medical Centre, Rotterdam, The Netherlands

**Keywords:** biomarker, breath test, electronic nose technology, gas chromatography-mass spectrometry, interstitial lung disease

## Abstract

There is a need for better noninvasive tools to diagnose interstitial lung disease (ILD) and predict disease course. Volatile organic compounds present in exhaled breath contain valuable information on a person's health and may be a novel biomarker in ILD. In this review, we will give an overview of the basic principles of breath analysis, summarize the available evidence in ILD, and discuss future perspectives.

**Recent findings:**

An increasing number of studies on exhaled breath analysis were performed over the last decade in patients with ILD, using two methods for exhaled breath analysis: gas chromatography-mass spectrometry and electronic nose technology. Most studies showed high accuracy for diagnosis of ILD, but study design and methods widely varied. Studies investigating the potential of electronic nose technology to predict treatment response and disease behavior are ongoing.

**Summary:**

The majority of studies using exhaled breath analysis in ILD show promising results for diagnostic purposes, but validation studies are lacking. Larger prospective longitudinal studies using standardized methods are needed to collect the evidence required for developing an approved diagnostic medical test.

## INTRODUCTION

Around 400 BC, Hippocrates already mentioned the importance of the human nose as diagnostic tool. He related the typical smell of various body secretions, like breath, sputum, urine and stool, to a certain diagnosis [1]. In the past few years, analysis of exhaled breath has increasingly been studied as potential diagnostic marker in a wide range of (respiratory) disorders, including interstitial lung disease (ILD) [2–4].

ILDs form a heterogeneous group of more than 200 different lung diseases in which the interstitium of the lung is affected by fibrosis, inflammation, or a combination of both [5]. Symptoms as dyspnea, cough, and fatigue are nonspecific, and there is no single noninvasive diagnostic test for ILD. Hence, delay during the diagnostic process and referral to specialized hospitals is common [6]. Therefore, better screening and diagnostic tools are needed. Disease course of different ILDs is highly variable and even within specific diagnoses, disease behavior and response to therapy varies between patients. This highlights the importance of new prognostic and predictive biomarkers. However to date, no reliable blood biomarkers have been found in ILD [7]. As exhaled breath provides additional information about a person's health status, this is an interesting new biomarker source for ILD.

Compared with ILD, exhaled breath analysis has more extensively been studied in other lung diseases, with lung cancer being the main area of research in the last years. Kort *et al.*[8] recently reported results from a multicenter validation study of breath analysis in lung cancer. The robust results on differentiating patients with and without lung cancer show the potential value of using eNose technology as a diagnostic tool in medical practice. Strikingly, eNose technology can accurately predict response to treatment in patients with stage 4 nonsmall cell lung carcinoma [9,10]. Validation studies are currently ongoing. More detailed information on eNose technology in other lung diseases can be found elsewhere [4].

In this review, we will focus on the potential of exhaled breath analysis in ILD, describe basic principles of different analysis methods, summarize available evidence in patients with ILD, and discuss future perspectives of exhaled breath analysis in ILD. 

**Box 1:**
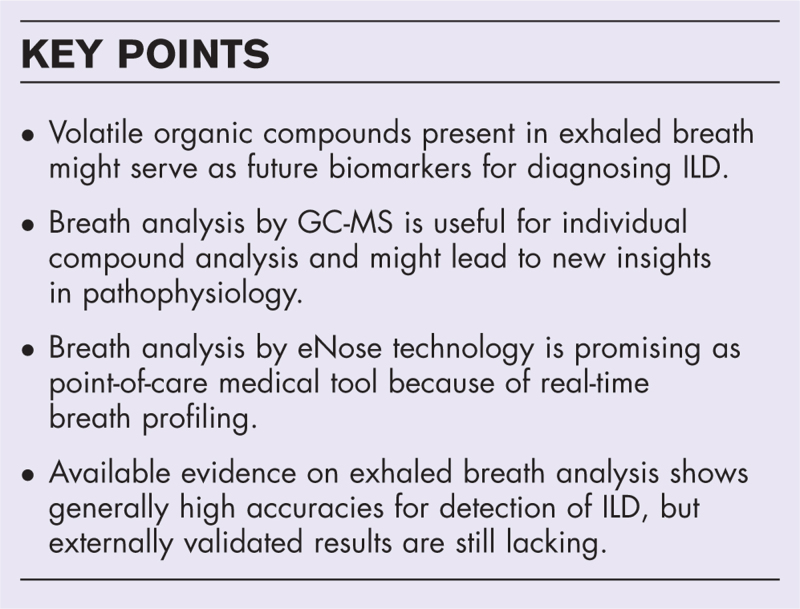
no caption available

## EXHALED BREATH ANALYSIS

Exhaled breath contains different types of compounds from exogenous and endogenous origin. Compounds range from large (e.g. microorganisms) to smaller compounds. The smaller compounds can be categorized as volatile (i.e. evaporates easily) or nonvolatile, and as organic (i.e. contains carbon) or nonorganic. For each category, different breath sample and analysis methods are required to capture the compounds. An overview can be found in Table 1.

**Table 1:** Examples of collection and analysis methods of exhaled breath compounds

Target compound	Example	Breath sampling	Breath analysis
Nonvolatile organic compounds and water-soluble volatile molecules	Lipids, amino acids	Exhaled breath condensate	Spectrometry or enzyme immunoassay
Volatile organic compounds	Acetone, ethanol	Exhaled air^a^	Spectrometry or (cross-reactive) sensors
Volatile nonorganic compounds	Nitric oxide	Exhaled air^a^	Specific sensor

This table includes the most common ways of sampling and analyzing breath, and is not intended being a complete overview as no standard approach exists.

a Exhaled air can be collected and processed in a sampling bag prior to compound analysis or can be captured and stored directly by exhaling through a device.

Especially, the analysis of volatile organic compounds (VOCs) is of interest in biomarker research. The concentration and type of VOCs (i.e. the volatilome) are affected by various (patho)physiological processes in the body and are unique for all individuals. The majority of endogenous VOCs originate from metabolic activity of organs or human microbiota, and from pathologic processes [11]. Subsequently, VOCs are excreted to the blood stream, diffused to and exhaled via the alveoli, or excreted by other organs such as the gut, kidneys or skin. As breath is the main source of VOCs and the lung tissue itself also excretes VOCs, breath analysis is mostly studied in respiratory diseases [11,12].

**FIGURE 1:**
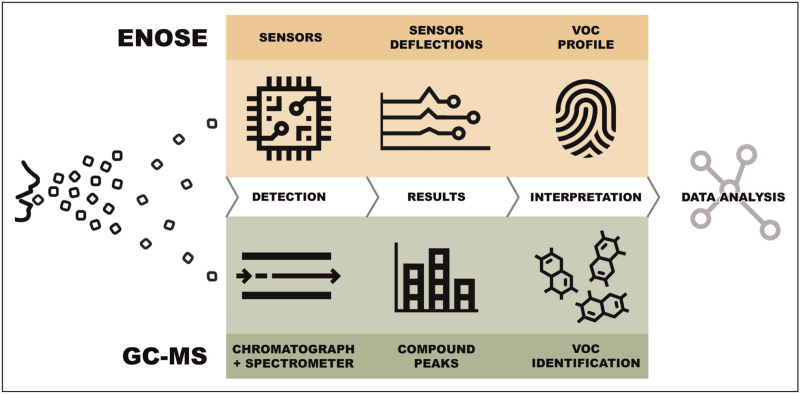
Overview and comparison of gas chromatography combined with mass spectrometry and electronic nose breath analysis. eNose, electronic nose; GC-MS, gas chromatography combined with mass spectrometry; VOC, volatile organic compounds.

Researchers can either choose a targeted or nontargeted approach when analyzing VOCs in breath. A targeted approach is hypothesis-based and aims to identify one or more predefined VOCs. Nontargeted analysis looks for differentiating VOCs or patterns in the full volatilome without prior knowledge or assumptions. This nontargeted approach is often called ‘breathomics’, as it shares similarities with the field of genomics, proteomics and metabolomics. In general, two different methods can be used to analyze VOCs: gas chromatography-mass spectrometry (GC-MS) or a sensor-based technique (so-called electronic nose, eNose). GC-MS analysis can be either targeted or nontargeted, but eNose research follows a nontargeted approach. Figure 1 shows a schematic overview of similarities and differences of these methods.

## GAS CHROMATOGRAPHY-MASS SPECTROMETRY

The use of GC-MS to analyze VOCs in exhaled breath originates from the 1970s [13]. This analytical method combines two steps to identify compounds in gas mixtures. In short, during gas chromatography, gaseous compounds are separated into molecules by sending the breath sample through a capillary column. All molecules leave the column at different times, resulting in a specific retention time. Subsequently, a mass spectrometer is used to ionize the molecules and calculate a mass-to-charge ratio of ionized molecules. The ratio can be used to identify specific VOCs by comparison with mass spectral libraries. Results are usually presented in a chromatogram, showing intensity peaks to indicate the concentration of all detected compounds. Technical and analytical variations exist for each step of GC-MS [14].

In the medical field, GC-MS could especially be useful for two purposes. First, this analysis method allows to identify individual compounds of exhaled breath, which might unravel pathophysiological processes. Second, many GC-MS studies evaluate the potential of specific VOCs as a new biomarker to diagnose or monitor specific conditions.

### Gas chromatography-mass spectrometry in interstitial lung disease

To date, only a small number of studies evaluated whether GC-MS analysis can detect ILD (Table 2). The first small pilot study in sarcoidosis compared VOC profiles of patients with those suspected of sarcoidosis. Suspected sarcoidosis was defined as the presence of enlarged mediastinal lymph nodes, without a confirmed diagnosis of sarcoidosis. There seemed to be differences between breath profiles of the two groups based on 13 discriminative chromatogram peaks. However, the authors only provided visual plots and did not perform statistical tests to evaluate whether breath profiles of the two groups were actually significantly different [15]. In 2017, a larger study found differences in sarcoidosis VOC profiles compared with healthy controls [16]. In both studies, not all patients had lung parenchymal involvement.

**Table 2:** Main results of volatile organic compound breath analysis in patients with interstitial lung disease using chromatography and spectroscopy

Author	Year	Patient groups (n=)	Technique	Comparison	Discriminative compounds (n=)	Performance (AUC/accuracy)
Plantier *et al.*[19^▪▪^]	2022	IPF (53) CTD-ILD (51) HC (51)	GC-tof-MS	IPF vs. HC CTD-ILD vs. HC IPF vs. CTD-ILD	34 11 16	0.91/84.6% 0.84/77.5% 0.84/76.9%
Yamada *et al.*[20]	2017	IPF (40) HC (55)	MCC-IMS	IPF vs. HC	5	-/76.8–83.2%^a^
Yang *et al.*[17]	2017	Pneumoconiosis (25) Stone workers (154)	GC-MS	Pneumoconiosis vs. exposed	9	0.90/– ^c^
Fijten *et al.*[16]	2017	Sarcoidosis (87)^b^ HC (26)	GC-tof-MS	Sarcoidosis vs. HC	9	0.76/74.1%^c^
Jalali *et al.*[18]	2016	Silicosis (4) HC (45)^d^ Silica exposed (20)	GC-MS	Silicosis vs. exposed Silicosis vs. HC	Multiple results	Not reported
Westhoff *et al.*[15]	2007	Sarcoidosis (5)^e^ Sarcoidosis suspected (4)^f^	MCC-IMS	Sarcoidosis vs. suspected	13	No statistical test results reported
Hayton *et al.*[21]	*2020*	*IPF (46)*	*GC-MS*	*Stable vs. disease progression at 6 months*	*1*	*N/A*
Guiot *et al.*[22]	*2020*	*SSc (27, of which 17 with SSc-ILD)*	*Unknown*	*SSc-ILD vs. SSc without ILD*	*0*	*N/A*

Main results of the cited articles are displayed. Conference abstracts are shown in italic.

a A separate accuracy for each of the discriminative VOCs was calculated. AUC, area-under-the-curve; CTD, connective tissue disease; GC, gas chromatography; HC, healthy control; ILD, interstitial lung disease; IMS, ion mobility spectrometry; IPF, idiopathic pulmonary fibrosis; MCC, multicapillary column (i.e. variation of a capillary gas chromatograph); MS, mass spectrometry; SSc, systemic sclerosis; tof, time of flight (i.e. type of mass analyzer).

b *n* = 18 had Scadding stage 0.

c Results of test/validation cohorts or cross-validation analyses are displayed here.

d Group was split in 20 nonsmoking and 25 smoking individuals.

e Not all patients had ILD.

f Sarcoidosis excluded after biopsy of mediastinal lymphadenopathy.

Two studies were conducted in patients with occupational lung diseases. Yang *et al.*[17] studied stone workers with and without a pneumoconiosis diagnosis. Jalali *et al.*[18] included individuals exposed to silica, either with or without a diagnosis of silicosis. Both studies identified several VOCs that differentiated patients with ILD from exposed patients without ILD, but it is unclear whether these VOCs were overlapping.

A recent study showed differences in breath profiles of patients with idiopathic pulmonary fibrosis (IPF) and connective tissue disease (CTD)-associated ILD using GC-MS analysis. Breath profiles of the patient groups differed significantly, with 16 discriminative VOCs being identified [19^▪▪^]. This was the first breathomics study using GC-MS indicating that VOC profiles in pulmonary fibrosis depend on the underlying condition. However, no test or validation cohort was applied, so further research should elucidate whether results can be replicated and validated. Additionally, this article described 34 discriminatory VOCS between patients with IPF and healthy controls, of which five VOCs were most contributing. These five VOCs were different from the four identified significant VOCs detected by Yamada *et al.*[20] in a similar analysis between IPF and healthy individuals conducted in 2017. Several factors could have contributed to this discrepancy, including differences in methodology (e.g. breath collection, breath and data analysis methods) and included patient cohorts (e.g. sample size, patient characteristics and matching of controls). Alternatively, these results may be exemplary for the limited performance of individual VOCs as disease-specific biomarkers in ILD.

Preliminary data from conference abstracts during the last 3 years reported on new applications of GC-MS, such as prediction and screening. In a longitudinal cohort of patients with IPF, one specific VOC predicted disease progression after 6 months [21]. A study in patients with systemic sclerosis evaluated whether GC-MS analysis could be used for early detection of ILD in patients with systemic sclerosis. However, in this small cohort, there were no differences in VOCs between systemic sclerosis patients with or without ILD [22].

## ELECTRONIC NOSE TECHNOLOGY

eNose technology is a sensor-based technique for gas analysis based on the mammalian olfactory system. Exhaled VOCs are captured by an eNose device that contains multiple sensors (similar to the olfactory receptors in a human nose). These sensors have different sensitivities for ranges of VOCs, leading to specific sensor deflections that are subsequently pooled and processed to create a breath profile (Figure 1). By analyzing breath data with pattern recognition algorithms, specific diseases can be distinguished, as previously shown by eNose studies in a wide range of respiratory and nonrespiratory diseases [4,23,24^▪▪^]. The most important difference with GC-MS is that eNoses do not identify individual VOCs. Consequently, the purpose of eNose breath analysis is not to elucidate disease pathophysiology but rather to use as a point-of-care diagnostic tool in clinical practice.

The first eNose was developed in 1964, but it was not until the 1980s that the first studies on the use of eNose in the medical field were published, and that the term electronic nose was used for the first time [25]. Since then, eNose technology has received increasing attention, and a variety of eNose devices has been developed and is currently available on the market for research purposes. These devices differ in type and number of sensors (electrical, gravimetric and optical sensors), portability, method of breath collection (e.g. direct online analysis, or collection and storage on-site), correction for ambient air or other possible confounders, and technology readiness level [4]. To our knowledge, there are no studies available that directly compare the performance of different eNose devices, and hence, the choice for a device may depend on research setting, costs, and availability.

### Electronic nose technology in interstitial lung disease

Several single-center studies on the potential of eNose technology for identification of ILDs have been published over the last 10 years (Table 3). In these studies, different patient populations, eNose devices, and analysis techniques have been used. The first small pilot study in 2013 found that breath profiles of patients with untreated pulmonary sarcoidosis differed from healthy controls, with a cross-validated accuracy of 83.3% [26]. However, breath profiles of patients receiving immunosuppressive medication for sarcoidosis could not be distinguished from healthy controls. This implies that inflammation influences the breath profile in patients with sarcoidosis, as adequately treated patients were less likely to have ongoing inflammation. The potential of eNose technology to separate patients with sarcoidosis from healthy controls was confirmed by a larger single-center study using a different type of eNose [27^▪^]. In this cohort, there was 100% discrimination between patients with sarcoidosis and healthy controls, in both a training and test set, irrespective of the use of immunosuppressive medication and organ involvement. Patients with pulmonary sarcoidosis were adequately distinguished from patients with other ILDs, and in particular from patients with hypersensitivity pneumonitis, which is also characterized by granulomatous inflammation. External validation studies should further assess the ability of eNose to differentiate sarcoidosis from other granulomatous diseases. Within the group of patients with sarcoidosis, there were no distinctive differences in breathprint, except between patients with a normal and elevated serum-soluble IL2 receptor level. As the soluble IL2 receptor is a marker for inflammatory activity in sarcoidosis, this result also suggests an influence of systemic inflammation on breath profiles.

**Table 3:** Main results of volatile organic compound breath analysis in patients with interstitial lung disease using electronic nose technology

Author	Year	Patient groups (n=)	eNose device	Comparison	Performance (AUC/accuracy)
Van der Sar *et al.*[27^▪^]	2022	Sarcoidosis (252, of which 224 pulmonary) ILD (317, of which 50 HP) HC (48)	SpiroNose	Sarcoidosis vs. HC Pulmonary sarcoidosis vs. ILD Pulmonary sarcoidosis vs. HP	1.00/100%^a^ 0.87/83.2%^a^ 0.88/87.8%^a^
Xuan *et al.*[29^▪^]	2022	Silicosis (221, of which 85 stage I disease) Miners (398)	Customized system^b^	Silicosis vs. miners Stage I silicosis vs. miners	0.77–0.89/78.5–84.3% 0.78–0.94/70.8–91.7%
Moor *et al.*[30]	2021	ILD (215, of which 85 IPF) HC (48)	SpiroNose	ILD vs. HC IPF vs. non-IPF	1.00/100%^a^ 0.87/91%^a^
Dragonieri *et al.*[31]	2020	IPF (42) COPD (43) HC (46)	Cyranose 320	IPF vs. HC IPF vs. COPD	1.00/98.5% 0.85/80.0%
Krauss *et al.*[32]	2019	ILD (174, of which 51 IPF and 25 CTD-ILD) COPD (23) HC (33)	Aeonose	IPF vs. HC CTD-ILD vs. HC IPF vs. CTD-ILD CTD-ILD vs. COPD	0.95/- 0.90/– 0.84/– 0.85/–
Yang *et al.*[28]	2017	Pneumoconiosis (34) Stone workers (64)	Cyranose 320	Pneumoconiosis vs. workers	0.86–0.89/65.0–70.0%^a^
Dragonieri *et al.*[26]	2013	Pulmonary sarcoidosis (31, of which 11 untreated) HC (25)	Cyranose 320	Sarcoidosis untreated vs. HC Sarcoidosis untreated vs. treated	0.83/83.3% –/74.2%
Van der Sar *et al.*[34]	*2022*	*ILD (42, of which 22 starting immunosuppressive and 20 antifibrotic treatment)*	*SpiroNose*	*Yes vs. no response to immunosuppressants* *Yes vs. no response to antifibrotics*	*0.84/*– *0.75/*–
Van der Sar *et al.*[33]	*2021*	*Pulmonary fibrosis (304)*	*SpiroNose*	*N/A (unsupervised analysis)*	*3 distinct clusters identified*

Main results of the cited articles are displayed. Conference abstracts are shown in italic. Displayed size of patient groups (n) are the sum of training and test/validation cohorts, if applicable. AUC, area-under-the-curve; COPD, chronic obstructive pulmonary disease; CTD, connective tissue disease; eNose, electronic nose; HC, healthy control; HP, hypersensitivity pneumonitis; ILD, interstitial lung disease; IPF, idiopathic pulmonary fibrosis.

a Results of independent test/validation cohorts.

b Based on Pilot (Vaporsense) sensor array.

The potential of eNose technology in pneumoconiosis has been assessed in two studies [28,29^▪^]. Yang *et al.*[28], who also studied GC-MS in this population, found a relatively high area under the curve (AUC) for differentiating patients with pneumoconiosis from a control group of stone workers. A larger study published in 2022 evaluated breath profiles in a cohort of miners, with and without silicosis [29^▪^]. Their customized eNose system showed a good accuracy in a training and an external validation set, also for patients with early-stage disease. A strength of these studies is that they compared breath data of patients with a cohort at-risk for developing pneumoconiosis, suggesting that eNose technology has potential as a screening tool in this population.

Three research groups, each using a different eNose, showed that the breath profile of patients with IPF could be very well discriminated from healthy controls [30–32]. The first study from 2019 also showed a high accuracy when comparing CTD-ILD with healthy controls. Nevertheless, the accuracy to detect differences within the group of ILDs was slightly lower, and data were not validated [32]. A large single-center study found that patients with IPF had significantly different breath profiles than patients with other forms of pulmonary fibrosis (accuracy 91%, confirmed in a test set) [30]. There were also distinctive differences between individual ILDs, but group sizes were small, and results need external validation. Dragonieri *et al.*[31] found an accurate distinction between IPF and COPD in a training and external validation cohort, and a significant correlation between total cell count in bronchoalveolar lavage and eNose sensor data. The current available data imply that eNose technology can be used as a noninvasive tool for screening and diagnostic purposes: to distinguish ILD from other chronic respiratory diseases and to classify and phenotype individual ILDs.

An exploratory study, of which results were presented as conference abstract in 2021, analyzed the potential of unsupervised analysis in a pulmonary fibrosis cohort [33]. In a group of 304 patients, three different clusters could be identified based on breath profiles. Clusters significantly differed with regard to diagnosis, gender, and immunosuppressant use, again indicating that breath profiles are influenced by inflammation. Longitudinal follow-up is needed to evaluate whether these clusters are associated with disease behavior and progression. Another application of eNose data is the prediction of disease behavior. A study in a small cohort of ILD patients suggested that eNose technology has the potential to predict treatment response in patients before starting on antifibrotic treatment (AUC 0.75) and immunosuppressive treatment (AUC 0.84) [34].

## FUTURE CHALLENGES AND PERSPECTIVES

The summarized evidence in this review shows that VOCs in exhaled breath hold valuable information for diagnosing ILD and potentially for prediction of disease course in individual patients. eNose breath tests hold great promise as a noninvasive, quick, and relatively low-cost medical application for ILD. Further validation in different cohorts and other important challenges need to be addressed before current research findings can be translated into an approved and validated medical test.

To date, there are no breath analysis studies in ILD published that replicate and validate previous findings in new patient cohorts. Moreover, available results are difficult to compare, which is partly because of differences in study design or healthcare setting. Many different methods and devices exist for breath collection and processing, VOC identification or VOC profile creation, and data analysis. Validation studies with new patient cohorts following similar standardized procedures are highly needed to test and validate various GC-MS and eNose applications.

GC-MS has already been studied for decades, but this technique has not made it to clinical practice in any medical field yet. There might be several reasons for this. Breath analysis with a chromatograph and spectrometer is a complex technique. The procedure is precise, elaborative and requires experienced investigators. Many labs have their own methods for breath collection and analysis, and approach to correct for possible confounders such as ambient air, environment or patient-related factors. Another reason why GC-MS studies have failed in finding a reliable biomarker for ILD might be that studies mainly focus on a combination of one or more significant individual VOCs. Single VOCs can provide valuable information on pathophysiological processes but can be influenced by various endogenous or exogenous factors that are difficult to identify or eliminate. Therefore, an approach that identifies a breath profile rather than individual VOCs may be more suitable when aiming to find a biomarker or medical test [2]. eNose technology has the advantage of creating this breath profile instantly by combining multiple sensor deviations. Besides, compared with GC-MS, measurements are less time-consuming and easier to perform. Moreover, there is immediate feedback on the measurement quality when using a device connected to an online platform. An online device facilitates analysis of breath data in real-time, which makes eNose technology suitable as point-of-care medical test. Especially when an eNose device corrects for known confounders, such as ambient air, it can be expected that findings can be replicated in various locations and healthcare settings.

**FIGURE 2:**
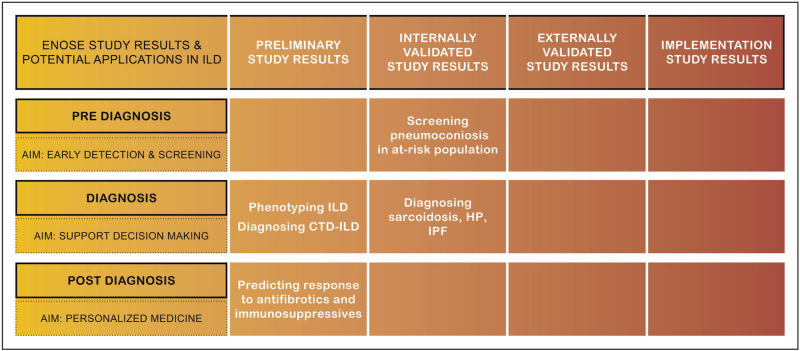
Overview of available evidence on electronic nose technology in interstitial lung disease for each research step towards clinically applicable breath tests. Evidence is categorized per phase with corresponding applications within the patient journey (before, during or after the diagnostic phase). No studies published externally validated data or implementation study data. CTD, connective tissue disease; HP, hypersensitivity pneumonitis; ILD, interstitial lung disease; IPF, idiopathic pulmonary fibrosis.

Until now, the majority of exhaled breath studies in ILD focused on differentiating patient groups, to develop a diagnostic tool for ILD. Data on other applications as disease phenotyping, prediction of disease course, or response to treatment are preliminary. Figure 2 shows an overview of the current status of developing clinical breath tests for ILD, with evidence from eNose studies in ILD categorized by phase of the diagnostic trajectory and clinical application. This figure highlights that none of the outcomes in ILD are externally validated and no implementation studies have been performed yet. To collect robust evidence for a clinically applicable breath test, all research steps need to be completed for each specific application and individual ILD diagnosis. To make this process more efficient and less costly, we need multinational collaboration in large research projects. An ongoing multicenter longitudinal trial in four European countries will evaluate diagnostic accuracy for individual ILDs and assess the value of eNose technology as biomarker for disease progression and response to treatment (NCT04680832).

The ultimate future diagnostic breath test would profile the full human volatilome in real-time following a standardized procedure, correct for confounders, and be connected to an online database. The output of this test could be a probability score of individual ILD diagnoses for a particular patient (e.g. 85% probability that this patient has IPF) to support decision-making by physicians and multidisciplinary team discussions. Such a test might prevent invasive procedures in the diagnostic work-up of patients. A breath test using eNose technology is likely to be more suitable for this purpose than GC-MS. Nevertheless, comparison with GC-MS data might be of additional value to gain more insights in pathophysiological processes, and for the calibration or optimization of the medical test.

## CONCLUSION

Since Hippocrates alluded to the nose as important diagnostic tool more than 2000 years ago, different techniques have been developed for exhaled breath analysis. Studies on eNose technology in ILD showed promising results for various clinical applications in ILD, but its value as a diagnostic and prognostic biomarker should be further explored and validated in the upcoming years.

## Acknowledgements

*None.*

### Financial support and sponsorship

*None.*

### Conflicts of interest

*I.S. reports that her research is supported by an unrestricted grant from Boehringer-Ingelheim paid to Erasmus Medical Center. C.M. reports an unrestricted research grant from Boehringer-Ingelheim, Astra-Zeneca and Daiichi-Sankyio, paid to Erasmus Medical Center. M.W. reports received consultancy or speaker fees from Astra-Zeneca, Bristol Myers Squibb, CSL Behring, Galapagos, Galecto, Horizon Therapeutics, Kinevant Sciences, Molecure, NeRRe Therapeutics, Novartis, PureTech Health, Thyron and Vicore paid to Erasmus Medical Center; and grants from Boehringer-Ingelheim, Astra-Zeneca/Daiichi-Sankyo and Hoffmann-La Roche outside the submitted work.*

## REFERENCES AND RECOMMENDED READING

Papers of particular interest, published within the annual period of review, have been highlighted as:

▪ of special interest
▪▪ of outstanding interest
